# Absence of Association between -1131T>C Polymorphism in the Apolipoprotein APOA5 Gene and Pediatric Metabolic Syndrome

**Published:** 2014-06

**Authors:** Sayedeh Ghazaleh Fatemi, Modjtaba Emadi-Baygi, Parvaneh Nikpour, Roya Kelishadi, Mahin Hashemipour

**Affiliations:** 1 Department of Genetics, School of Basic Sciences; 2 Research Institute of Biotechnology, School of Basic Sciences, Shahrekord University, Shahrekord; 3 Pediatric Inherited Diseases Research Center, Isfahan University of Medical Sciences, Isfahan; 4 Department of Genetics and Molecular Biology, School of Medicine; 5 Child Growth and Development Research Center, Isfahan University of Medical Sciences, Isfahan, Iran

**Keywords:** Apolipoprotein A5 Gene; Metabolic Syndrome; Children; Adolescents; Triglyceride; Cholesterol

## Abstract

***Objective:*** In the present study, we evaluated the association of rs662799 variant of the *APOA5* gene with Metabolic syndrome (MetS) in a sample of children and adolescents from Isfahan.

***Methods:*** This case control study comprised 50 cases of MetS and 50 controls. Mismatched polymerase chain reaction–restriction fragment length polymorphism (mPCR-RFLP) was used to genotype -1131T>C polymorphism.

***Findings*** ***:*** No significant association was documented for *APOA5* genotypes with the measured laboratory parameters for CC, CT, and TT genotypes in the two groups studied. By logistic regression using a dominant model, the odds ratio (95% confidence interval0 for the MetS was 0.38 (0.139–1.0350 and 0.29 (0.08–1.071 for the unadjusted and adjusted models, respectively.

***Conclusion:*** This study suggests that among studied children and adolescents, -1131T>C polymorphism in the *APOA5* gene may not be a major contributor to the MetS risk.

## Introduction

Metabolic syndrome (MetS) is a prevalent complex disorder consisting of concurrent metabolic abnormalities^[^^1^^]^. The prevalence of the syndrome is 1-2% in Iranian children and adolescents, much higher than that reported for other ethnicities^[^^2^^-^^4^^]^. It markedly increases the risk of developing cardiovascular diseases and type 2 diabetes^[^^5^^]^. According to the ATPIII criteria, MetS can be diagnosed based on hypertension, central obesity, insulin resistance and dyslipidemia. However, the most frequent components of the MetS in Iranian children and adolescents are low high-density lipoprotein cholesterol (HDL-C) and high triglycerides (TG)^[^^2^^]^.

 Among the genetic variants associated with the development of MetS, there is a naturally occurring variant (-1131T>C) in the promoter region of apolipoprotein A5 gene (*APOA5*). The mature APOA5 protein expresses exclusively in the liver and secretes into the plasma to modulate TG metabolism^[^^6^^]^.

 The -1131T>C (rs662799) variant of *APOA5* gene is associated with increased triglyceride levels and confers risk for metabolic syndrome in adult populations^[^^7^^-^^13^^]^. However, little is known about the *APOA5* variants in pediatric MetS. In the present study, we evaluated the association of rs662799 variant of the *APOA5* gene with MetS in Isfahanian children and adolescents.

## Subjects and Methods

A total of 50 cases of MetS and 50 controls were recruited to this study. Controls had normal weight and were healthy looking, without any clinical, laboratory or history records for MetS, diabetes or cardiovascular disorders. To diagnose the individuals with MetS, we used the modified ATPIII definition^[^^2^^]^. In brief, a MetS subject fulfilled at least three of the following components: waist circumference >75^th^ percentile for age and gender in the studied population; fasting TG ≥100mg/dl; HDL-C <50 mg/dl (except in 15-19 year boys in whom the cut off was <45 mg/dl); systolic blood pressure/diastolic blood pressure >90^th^ percentile recommended cut off points by the National Heart, Lung and Blood institute for gender, age and height^[^^14^^]^; fasting blood sugar (FBS) ≥100 mg/dl^[^^2^^]^. This study was approved by the Ethic Committee of Isfahan University. Informed consent was obtained from parents. The experimental design was approved by the Ethics Committee of Shahrekord University.

 Blood specimens were collected in EDTA-treated tubes and were stored at –70°C for further analysis. Extraction of DNA from total blood was performed using the Diatom DNA Prep 100 kit (Isogen Laboratory, Russia). mPCR-RFLP was used to genotype T>C polymorphism with primers described elsewhere^[^^7^^]^. The amplified fragment possessed an obligatory cleavage site for *Tru*I restriction endonuclease to check if the digestion has occurred. Thermal conditions of the PCR cycles were as follow: initial denaturation at 95°C followed by 35 amplification cycles consisting of denaturation at 95°C for 40 sec; annealing at 61°C for 40 sec; extension at 72°C for 40 sec and a final extension at 72°C for 10 min. 10 μl of the PCR product was digested with 5 U of *Tru*I enzyme. According to the digestion patterns, three genotypes were determined: TT genotype resulted in 21, 108 and 267 bp fragments, TC genotype created 21, 108, 267 and 288 bp products and CC genotype produced 108 and 288 bp fragments.

 Statistical analyses were done with SPSS software (version 20.0). *P* values less than 0.05 were considered significant. A Chi-square statistic was calculated to compare the frequencies of genotypes and alleles. One way ANOVA was conducted to evaluate any differences between different groups, regarding the levels of biochemical factors and the distribution of different genotypes. Logistic regression analysis was performed to derive the odds ratios.

***Findings***

The major relevant clinical and biochemical characteristics of the study participants are presented in Table 1. The main risk factors for MetS were significantly augmented in the MetS cases except for HDL-C (*P*<0.01). 

**Table 1:** Clinical and biochemical data of the MetS and control subjects

**Parameter**	**Cases (n=50)**	**Controls (n=50)**	***P*** ** value**
**Boys/Girls**	22(44%)/28(56%)	25(50%)/25(50%)	0.5
**Body mass index (kg/m** ^2^ **)**	26.4 (0.45)	17.85 (0.94)	<0.001
**Age (years)**	12.18 (0.24)	13.48 (0.44)	0.01
**Triglyceride (mg/dl)**	109.64 (8.24)	78.44 (5.12)	0.002
**Total Cholesterol (mg/dl)**	165.12 (3.80)	151.48 (14.14)	0.02
**High Density Lipoprotein-C (mg/dl)**	43.08 (0.74)	50.44 (1.74)	<0.001
**Low Density Lipoprotein -C (mg/dl)**	95.66 (3.32)	79.32 (1.88)	<0.001
**Fasting Blood Suger (mg/dl)**	102.56 (1.35)	91.24 (1.77)	<0.001

**Table 2:** Biochemical factor levels in MetS and control subjects according to the *APOA5* -1131T>C genotypes

**Biochemical ** **factors**	**Cases**		***P. *** **value**	**Controls**		***P. *** **value**
	**TT (n=43)**	**TC+CC (n=7)**		**TT (n=35)**	**TC+CC (n=15)**	
**TG (mg/dl)**	111.93 (59.55)	95.57 (51.33)	0.2	77.37 (36.14)	80.93 (37.59)	0.4
**TC (mg/dl)**	164.05 (24.99)	171.71 (38.74)	0.2	152.51 (30.52)	149.07 (27.07)	0.3
**HDL-C (mg/dl)**	43.37 (4.99)	41.29 (5.67)	0.2	49.97 (13.39)	51.53 (9.97)	0.3
**FBS (mg/dl)**	102.30 (8.64)	104.14 (15.08)	0.3	91.60 (14.51)	90.40 (6.35)	0.4
**LDL-C (mg/dl)**	93.93 (21.41)	106.29 (33.93)	0.1	80.71 (12.56)	76.07 (14.80)	0.1

TG: Triglyceride; TC: Total cholesterol; HDL-C: High density lipoprotein-cholesterol; LDL-C: Low density lipoprotein-cholesterol; FBS: Fasting blood sugar

Table 2 shows the comparison of the laboratory parameters of the control and MetS subjects stratified based on rs662799 genotypes. The data demonstrated that there was no evidence of an association of *APOA5* genotypes with any of laboratory parameters. Genotype and allele frequencies are shown in Table 3. The data revealed that the frequency of C allele was greater in controls compared with that of the MetS. The odds ratio of the C allele in MetS versus control subjects in the unadjusted model was OR=0.38, 95%CI: 0.14-1.03, *P*=0.05. The Odds ratio adjusted for sex, age, HDL-C and TC was also calculated (OR=0.29, 95%CI: 0.08-1.07, *P*=0.06) and no significant differences between adjusted and unadjusted models were found (Table 4).

## Discussion

We did not find an association between the -1131C polymorphism and elevated triglyceride levels. A number of studies have reported a significant association between triglyceride levels and the -1131T>C variant in MetS adults and obese children^[^^7^^-^^10^^,^^15^^-^^18^^]^. Our finding was in agreement with Mattei 's^[^^19^^]^ results which showed no association between TG levels and the -1131T>C.

**Table 3:** Genotype and allele frequencies

**Group**		**Cases ** **(n=50)**	**Controls ** **(n=50)**
**Allele ** **frequencies**	**C**	6 (12%)	9 (18%)
	**T**	44 (88%)	41 (82%)
**Genotype ** **frequencies**	**TT**	43 (86%)	35 (70%)
	**TC**	2 (4%)	12 (24%)
	**CC**	5 (10%)	3 (6%)

This inconsistency may be due to ethnicity influences^[^^20^^]^. Moreover, no association was found between the -1131T>C and MetS in our study. Significant correlation of -1131T>C* APOA5* variant with MetS in adults has been indicated in several^[^^8^^-^^13^^]^ but not all previous studies^[^^7^^,^^17^^-^^19^^]^. The findings of the current study are consistent with a recent meta-analysis^[^^21^^]^ which showed that C allele carriers of -1131T>C had overall a significantly higher risk of MetS. After performing subgroup analysis according to ethnicity, the association was only significant in Asians, but not in white populations.

## Conclusion

We showed that -1131T>C variant was neither associated with triglyceride levels nor MetS. Our results call for further studies to explore the effect of other *APOA5* SNPs and haplotypes in Iranian children and adolescents.

**Table 4:** Binary logistic regression analysis of the association between carrying *APOA5* -1131C allele and the risk for MetS

**Group**		**TT**	**TC+CC**
**Cases (n=50)**		43 (86%)	7 (14%)
**Controls (n=50)**		35 (70%)	15 (30%)
**Unadjusted ** **model**	**Odds ratio**	0.38	
	**95% CI**	0.14-1.03	
	***P.*** ** value**	0.05	
**Adjusted ** **model**	**Odds ratio**	0.29	
	**95%CI**	0.08-1.07	
	***P.*** ** value**	0.06	

CI: Confidence Interval

## Authors’ Contribution

S.G. Fatemi: Doing experiments, analysis of data, drafting the article. 

M. Emadi-Baygi: Conception, designing the study, analysis and interpretation of data the article. 

P. Nikpour: Conception, designing the study, analysis and interpretation of data and drafting and revising the article. 

R. Kelishadi: Sample collection, designing the study, analysis and interpretation of data the article. 

M. Hashemipour: Sample collection, analysis and interpretation of data the article. 

All authors approved final version of the manuscript.
